# Publisher Correction: Magnetic field enhancement of organic photovoltaic cells performance

**DOI:** 10.1038/s41598-018-21980-z

**Published:** 2018-03-13

**Authors:** S. Oviedo-Casado, A. Urbina, J. Prior

**Affiliations:** 1 Departamento de Física Aplicada, Universidad Politécnica de Cartagena, Cartagena, 30202 Spain; 2 Departamento de Electrónica, Universidad Politécnica de Cartagena, Cartagena, 30202 Spain

Correction to: *Scientific Reports* 10.1038/s41598-017-04621-9, published online 27 June 2017

Due to a typesetting error, Figure 1 in the original version of this Article was incorrect. This error has now been corrected in the PDF and HTML versions of the Article.

